# Diagnostic Accuracy of Holotranscobalamin, Vitamin B12, Methylmalonic Acid, and Homocysteine in Detecting B12 Deficiency in a Large, Mixed Patient Population

**DOI:** 10.1155/2020/7468506

**Published:** 2020-02-07

**Authors:** Araceli Jarquin Campos, Lorenz Risch, Urs Nydegger, Jacobo Wiesner, Maclovia Vazquez Van Dyck, Harald Renz, Zeno Stanga, Martin Risch

**Affiliations:** ^1^Universidad Autonòma de Guadalajara, Faculdad de Medicina, Zapopan, Jalisco, Mexico; ^2^Labormedizinisches zentrum Dr. Risch, Vaduz, Liechtenstein; ^3^Center of Laboratory Medicine, University Institute of Clinical Chemistry, University of Bern, Bern, Switzerland; ^4^Private University of the Principality of Liechtenstein, Triesen, Liechtenstein; ^5^Institute of Laboratory Medicine, Philipps University Marburg, Marburg, Germany; ^6^Department for Diabetes, Endocrinology, Nutritional Medicine and Metabolism, Bern University Hospital and University of Bern, Bern, Switzerland; ^7^Competence Centre of Military Disaster Medicine, Swiss Armed Forces, Ittigen, Switzerland; ^8^Zentrallabor, Kantonsspital Graubünden, Chur, Switzerland

## Abstract

Four biomarkers are commonly employed to diagnose B12 deficiency: vitamin B12 (B12), holotranscobalamin (HoloTC), methylmalonic acid (MMA), and homocysteine (Hcy). 4cB12, a combined index of the B12 status, has been suggested to improve the recognition of B12 deficiency. We aimed to evaluate the four different markers for detecting B12 deficiency, as determined by 4cB12. Within a large, mixed patient population, 11,833 samples had concurrent measurements of B12, HoloTC, MMA, and Hcy. 4cB12 was calculated according to the methods described by Fedosov. Diagnostic cutoffs as well as diagnostic accuracy for the detection of B12 deficiency were assessed with receiver operating characteristic (ROC) analysis. The median age was 56 years, and women accounted for 58.8% of the samples. Overall, the area under the curve (AUC) for the detection of subclinical B12 deficiency was highest for HoloTC (0.92), followed by MMA (0.91), B12 (0.9) and Hcy (0.78). The difference between HoloTC and B12 was driven by a significantly higher AUC for HoloTC (0.93) than for B12 (0.89), MMA (0.91), and Hcy in women 50 years and older (0.79; *p* < 0.05 for all). In the detection of subclinical B12 deficiency, there were no significant differences in the AUCs of HoloTC, B12, and MMA among men and women <50 years. In conclusion, in women < 50 years and in men, HoloTC, MMA, or Hcy do not appear superior to B12 for the detection of B12 deficiency. For women 50 years and older, HoloTC seems to be the preferred first-line marker for the detection of subclinical B12 deficiency.

## 1. Introduction

In human metabolism, vitamin B12 (B12; synonym: cobalamin) is an essential cofactor for two enzymes, methionine synthase and methylmalonyl-coenzyme A mutase [1, 2]. Thus, B12 is needed for myelin synthesis in the nervous system and, together with folate, is essential for the synthesis of DNA [1]. B12 deficiency occurs relatively frequently and can lead to the disturbance of myelin synthesis and to the prolongation of the S-phase in the cell cycle, causing increased cell volumes in a variety of cell types [1, 3, 4]. Clinically, B12 deficiency can be regarded as a symptom of different etiologies that affect a multitude of organ systems and biological functions [5]. Classically, the hematopoietic system (i.e., the bone marrow and peripheral blood) as well as the central and peripheral nervous systems is affected by a wide range of clinical sequelae (e.g., megaloblastic anemia, neurological symptoms, and neuropsychiatric disorders) [5, 6]. However, there are also other disorders associated with B12 deficiency, such as infertility and developmental delays or regression in children [1]. The neurological sequelae of B12 deficiency can be irreversible [7]. If treatment of the B12 deficiency is started early, most of the neurological symptoms will resolve, whereas delayed therapy will slow the progression of symptoms without ameliorating the symptoms [7, 8]. Thus, the early recognition and treatment of B12 deficiency is warranted [9].

When B12 deficiency is suspected, several circulating biomarkers can be used to confirm or dismiss the diagnosis [10]. These markers include two direct markers (i.e., total B12 and active B12, which is referred to as holotranscobalamin (HoloTC)) and two metabolic markers (i.e., homocysteine (Hcy) and methylmalonic acid (MMA)) [10–13]. The determination of total B12 relies on the integral measurement of two B12 fractions: the biologically active HoloTC (i.e., B12 bound to transcobalamin) and the biologically inactive holohaptocorrin (i.e., B12 bound to haptocorrin) fraction [2]. Total B12 concentrations can be nonspecifically affected by the altered expression of haptocorrin (e.g., during malignancy, pregnancy, liver disease, and autoimmune disorders) and by the analytical interference of antibodies [14–16]. Assays for HoloTC determinations only measure the biologically active B12 fraction, but this fraction may also undergo nonspecific alterations that affect its concentrations (i.e., transcobalamin polymorphisms, analytical interference, and B12 ingestion) [11, 17–22]. Hcy accumulates during B12 deficiency because of the diminished activity of methionine synthase, but Hcy can also increase due to folate deficiency, vitamin B6 deficiency, thyroid dysfunction, age, gender, and decreased glomerular filtration rate (GFR) [5]. MMA specifically accumulates in B12 deficiency due to the diminished activity of methylmalonyl-coenzyme A mutase and can also increase in response to decreased GFR [5]. Thus, each of these four markers of B12 deficiency is affected by factors that are not related to the B12 supply, leading to the decreased specificity of these markers [23]. Generally, MMA has been regarded by some authors as the most accurate marker [5], whereas HoloTC has been described as the earliest and most sensitive marker of B12 deficiency [22]. Nevertheless, since all four markers have nonspecific factors that influence their concentrations, it is generally acknowledged that there is no single best marker of B12 deficiency [13, 24].

Recently, Fedosov and colleagues described a combined index of B12 deficiency (4cB12) that integrates these 4 direct and metabolic markers [25–27]. This combined index was evaluated in a large collection of well-described cohorts and can be regarded as the most comprehensive marker of B12 deficiency, as this combined index has been described to be more reliable than any single biomarker [26, 27]. The use of this index has been proposed to improve the detection of subclinical B12 deficiency and to evaluate diagnostic cutoffs for single markers of B12 status [27, 28]. 4cB12 aids in the classification of B12 sufficiency into 5 different stages: elevated B12 (4cB12 > 1.5), B12 adequacy (4cB12 between -0.5 and 1.5), low B12 (4cB12 lower than -0.5 and higher than -1.5), possible B12 deficiency (4cB12 lower than -1.5 and higher than -2.5), and probable B12 deficiency (4cB12 lower than -2.5) [27]. For 4cB12 values lower than -0.5, supplementation with B12 should be considered [27].

With 4cB12, a novel combined potential reference index, especially for the subclinical stages of B12 deficiency, has become available [29]. Currently, little data is available on the suitability of these four individual markers to recognize the B12 status as defined by 4cB12 [11, 13]. The aim of the present study was to describe the cutoffs for the recognition of B12 deficiency with high specificity and sensitivity [27]. As a secondary aim, we investigated the diagnostic accuracy of the different markers to recognize the B12 status in a large, mixed patient cohort.

## 2. Materials and Methods

### 2.1. Study Population

**Figure 1:**
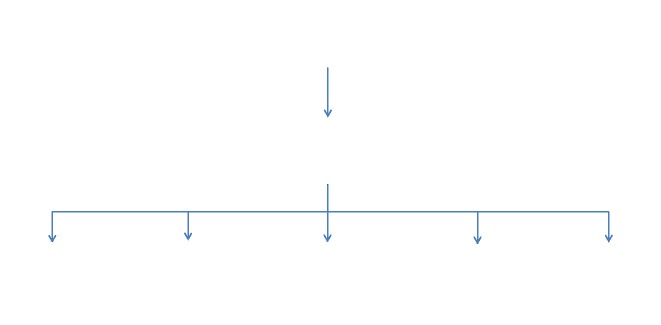
Flow chart of the sample inclusion. A total of 3,614 samples had a serum folate determination in addition to the results available from total B12, HoloTC, MMA, and homocysteine measurements.

In this retrospective cohort study, we analyzed our consecutive routine measurement results from investigations of the B12 status during the period from December 1, 2006, to October 19, 2018. From these routine measurements, we analyzed 887,871 clinical samples from patients referred for either the isolated or simultaneous determination of HoloTC, B12, Hcy, and MMA. Of these samples, a comprehensive assessment of the B12 status, including the simultaneous measurement of B12, HoloTC, MMA, and Hcy, was ordered for 11,833 samples. Indications for comprehensive testing were provided by the treating physicians and did not follow a standardized algorithm. A flow chart for the inclusion of samples is shown in Figure 1. The study protocol for the retrospective analysis of anonymized health data was verified by the cantonal institutional review board (KEK Bern), and the need for informed consent was waived (BASEC-Nr: Req-2018-00090).

### 2.2. Laboratory Methods

HoloTC, total B12, and homocysteine were assayed with commercially available immunoassays, whereas MMA has been measured by a LC/MS-MS system, as described elsewhere [30]. Measurements exceeding the upper limit of quantification (i.e., 128 pmol/L for HoloTC and 1,476 pmol/L for B12) were recorded as the next integer concentration value above the upper limit of quantification (i.e., 129 pmol/L for HoloTC, *n* = 1,388 and 1,477 pmol/L for B12, *n* = 251). The performance characteristics of these methods in our hands have been described in detail elsewhere, with CV's less than 9% for all markers (i.e., 6.6% for vitamin B12 (at a mean concentration of 162 pmol/L), 8.7% for folic acid in serum (18.9nmol/L), 6.8% for holoTC (46 pmol/L), 9.3% for Hcy (12.9 *μ*mol/L), and for 1.8% for MMA (390 nmol/L)) [4, 31, 32]. A combined indicator of the B12 status, 4cB12, was calculated as described by Fedosov and colleagues [27]. This method integrates the direct markers (HoloTC and B12) and metabolic markers (MMA and Hcy) of B12 deficiency and age based on models obtained from large empirical investigations. 4cB12 can be obtained according to the following equation:

(1) $$\begin{array}{c} 4\text{cB}12=\log_{10}\frac{\text{HoloTc}\times \text{B}12}{\text{MMA}\times \text{Hcy}}-\frac{3.79}{1+{\left(\text{age}/230\right)}^{2.6}}, \end{array}$$

where the direct markers (HoloTC and B12) are reported in pmol/L, and the metabolic markers (MMA and Hcy) are reported in *μ*mol/L. 4cB12≤−0.5 was defined as an indicator of low B12, with at least potential subclinical manifestations of B12 deficiency. A value<−1.5 indicates possible and probable B12 deficiency. The percentage of B12 bound to transcobalamin was calculated using the equation: 100 × (HoloTC/total B12) [20]. Haptocorrin-bound B12 was calculated by subtracting the HoloTC concentration from the total B12 concentrations [33].

### 2.3. Statistical Methods

Percentages are presented together with the 95% confidence interval (CI). Correlations were assessed with the Spearman rank method. Ratios were compared using a chi-square test. The diagnostic accuracy for the detection of subclinical (i.e., 4cB12<−0.5 and > -1.5) or potential or probable B12 deficiency (i.e., <-1.5) was calculated with receiver operating characteristic (ROC) curves. The cutoffs providing a 99% sensitivity and a 99% specificity as well as the optimum decision point (cut-off with least misclassification) for the different markers in detecting B12 deficiency were determined by ROC analysis. Then, ROC curves were separately analyzed for subgroups exploring age and gender differences. Administration of estradiol hormones and reduced kidney function has been shown to alter concentrations of markers of B12 status [5, 34, 35]. Because menopause occurs around age 50 and the prevalence of chronic kidney disease starts also to increase at age 50, we therefore chose to set the cutoff for a dichotomized age variable at 50 years (i.e*.,*age < 50 and age ≥ 50 years and samples from male and female patients) [36, 37]. The area under the curves (AUCs) were compared using a method described by Hanley and McNeil [38]. *p* values < 0.05 were considered statistically significant. In addition to an analysis of all the available samples, we further analyzed the cohort of patients only including the data from the first assessment of B12 status. The results of this second analysis is given in supplementary Tables [Supplementary-material supplementary-material-1][Supplementary-material supplementary-material-1]. The computer program Medcalc Version 18 (Medcalc Software bvba, Ostend, Belgium) was used to perform the calculations. The Standards for Reporting Diagnostic Accuracy Studies (STARD) 2015 guidelines were adopted for the present report [39].

## 3. Results

### 3.1. Baseline Characteristics, Correlations, and Cutoffs

A total of 11,833 samples from 9,464 patients were available for analysis. The samples were from patients with a median age of 56 years (interquartile range (IQR) (41, 68) years) and 6,991 (58.8%; 95% CI 57.9, 59.7) of these samples were from women. The concentrations of the serum markers, as well as 4cB12, are given in Table 1. 4cB12 was significantly correlated with Hcy (*r* = −0.5), MMA (*r* = −0.63), HoloTC (*r* = 0.8), and B12 (*r* = 0.8) (*p* < 0.001 for all). The cutoffs providing a 99% sensitivity and a 99% specificity as well as the optimum decision point for the different markers for the detection of subclinical B12 deficiency are provided in Table 2, whereas the respective cutoffs for the detection of possible or potential/probable B12 deficiency are provided in Table 3.

### 3.2. Overall Diagnostic Accuracy

**Figure 2:**
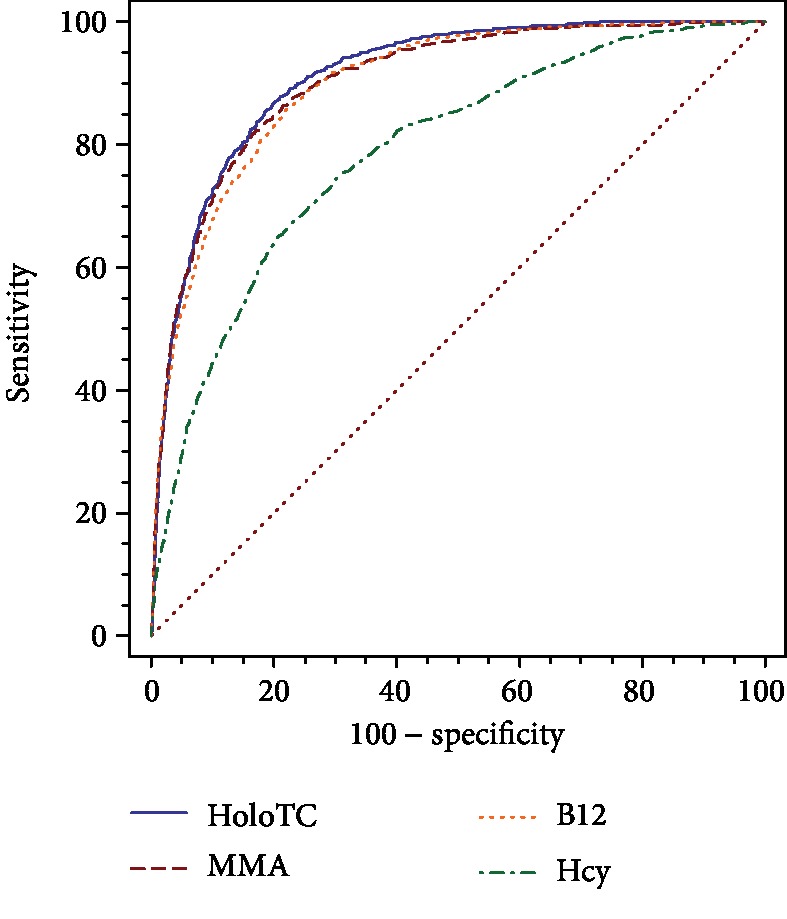
ROC curves for the four markers for detecting subclinical B12 deficiency (4cB12<−0.5).

Significant differences were observed among the diagnostic accuracy of the 4 markers (i.e., Hcy, MMA, HoloTC, and B12) for recognizing subclinical B12 deficiency (i.e., 4cB12<−0.5 and >-1.5). As shown in Figure 2, HoloTC had the highest diagnostic accuracy (AUC 0.912, 95% CI 0.907 to 0.917), followed by MMA (AUC 0.904, 95% CI 0.898 to 0.909), B12 (AUC 0.899, 95% CI 0.894 to 0.905), and Hcy (AUC 0.789, 95% CI 0.781 to 0.796). With the exception of MMA (*p* = 0.15), the differences between the AUC of the other markers were all significantly different from the AUC of HoloTC (*p* = 0.02 for B12, *p* < 0.001 for all other markers). The AUC of MMA was not significantly different from that of B12 (*p* = 0.47), whereas the AUC of Hcy was significantly lower than that of MMA (*p* < 0.001).

The diagnostic accuracy of the different markers for detecting a possible or probable clinical B12 deficiency (4cB12<−1.5) was then calculated. The diagnostic accuracies are ranked as follows: HoloTC (AUC 0.982, 95% CI 0.979 to 0.984), MMA (AUC 0.98, 95% CI 0.978 to 0.983), B12 (AUC 0.969, 95% CI 0.966 to 0.972), and Hcy (AUC 0.898, 95% CI 0.892 to 0.903). The AUC of MMA was not significantly different from that of HoloTC, (*p* = 0.92) or B12 (*p* = 0.41). The difference between HoloTC and B12 was also not significant (*p* = 0.23). The AUC of Hcy was significantly lower than that of the other 3 parameters (MMA, HoloTC, and B12; *p* < 0.01 for all).

Thus, HoloTC had the highest diagnostic accuracy for recognizing subclinical B12 deficiency (4cB12<−0.5) with significantly higher diagnostic accuracy than B12 and Hcy. In the case of possible or probable B12 deficiency, the diagnostic accuracy of HoloTC and the other markers was not significantly different.

### 3.3. Diagnostic Accuracy in Subgroups

Since diagnostic characteristics may change with age and gender due to hormonal changes or reduced kidney function, we further analyzed whether the diagnostic accuracy changed with these two factors. We divided the samples according to the patient gender and age (i.e., whether the person was at over or under 50 years old). These parameters resulted in 8 groups (i.e., women, men, individuals aged 49 and less, individuals aged 50 and over, women aged 49 and less, men aged 49 and less, women aged 50 and over, and men aged 50 and over). The diagnostic accuracies of HoloTC, B12, MMA, and Hcy to detect subclinical B12 deficiency (i.e., 4cB12<−0.5 and >-1.5) are given in Table 4. Hcy had a significantly lower diagnostic accuracy in detecting subclinical B12 deficiency than the other 3 markers. Further, the overall superiority of HoloTC to B12 and MMA seemed to be driven by the superior diagnostic performance of HoloTC in women aged 50 and over. A significant difference was not detected between the AUC of B12 and MMA in any of the subgroups. Identical trends were observed when the more severe levels of B12 deficiency were included in the analysis (i.e., 4cB12<−0.5, with additional samples exhibiting 4cB12<−1.5; data not shown).

However, as seen in Table 5, when evaluating the ability to recognize possible or probable B12 deficiency (i.e., 4cB12<−1.5), HoloTC showed the highest AUCs, but the values for HoloTC were not significantly different from those for the other markers (with the exception of Hcy). Thus, HoloTC seems to be superior to the other markers for detecting subclinical B12 deficiency in women 50 years and older. In women younger than 50 years of age and in men, HoloTC, MMA, and Hcy are not superior to B12 in detecting B12 deficiency.

## 4. Discussion

It is generally acknowledged that there is no single best marker for the diagnosis of B12 deficiency [40, 41]. Until the introduction of 4cB12 (which is currently the most expensive diagnostic option), MMA was thought to be the most sensitive but also the most expensive marker, while its specificity was debated, especially in elderly individuals [5, 13, 31, 37, 40]. The measurement of another metabolic marker, Hcy, is less complicated and is possible with high-performance liquid chromatography (HPLC) or immunoassays [42]. However, although Hcy is considered a sensitive marker, Hcy is substantially influenced by nonspecific factors that are unrelated to the B12 status (i.e., decreased GFR, vitamin B6 deficiency, and folate deficiency) [5, 10, 13]. The most widely used and least expensive parameter, i.e., B12, is often considered the least reliable marker of B12 deficiency, but it still reveals important information regarding the B12 status [10]. Finally, HoloTC has been described as an easily testable but costly marker of B12 deficiency, with only minor diagnostic advantages compared to B12 in clinical settings [11, 13, 22].

The value of HoloTC in subclinical B12 deficiency currently remains unclear because the diagnostic characteristics of HoloTC have not been independently evaluated with reference standard measurements that are better than MMA or red blood cell B12 [11, 13, 43]. The present study used a novel index to evaluate B12 sufficiency, i.e., 4cB12, and demonstrated that HoloTC is superior to B12 for recognizing subclinical B12 deficiency in a standard cohort, with approximately the same diagnostic accuracy as MMA. Subgroup analysis revealed that this superiority was driven by a clear superiority in women 50 years and older, while HoloTC was not superior to B12 in men and in women younger than 50 years. In women 50 years or older, HoloTC was superior to MMA and B12 for identifying B12 deficiency. Our results could help define an individualized screening strategy, in which B12 would be a first-line marker for men as well as for women younger than 50 years, whereas HoloTC would be the first-line marker for women 50 years and older.

With the use of 4cB12, the present study employed a reference for the diagnosis of B12 deficiency, which has only recently become available [25–27]. 4cB12 has been described as a more reliable diagnostic than any single biomarker, and it has the added benefit of allowing the detection of subclinical B12 deficiency [27]. 4cB12 provides a means of combining multiple pieces of information into one index, which has also been described to be of value for validating the conventional cutoff points of individual markers [27]. Clinical signs of B12 deficiency, such as reduced hemoglobin concentrations, cognitive function, and peripheral nerve conductivity, have been shown to be more strongly associated to 4cB12 than to the single markers that were used in other studies as reference markers. [5, 26]. Despite this observation and its superior performance, the 4cB12 index cannot yet be considered a universally accepted reference index [26].

When looking at the cutoffs to detect a low B12 status (i.e. 4cB12<−0 − 5 and >-1.5) with a high specificity (Table 2), these seem to be well in agreement with commonly employed cutoffs reviewed elsewhere [44, 45]. With the exception of Hcy, they fall in between the bands for the respective cut points described by Fedosov et al. (i.e., 20-37 pmol/L for HoloTC, 110-186 pmol for B12, 350-840 nmol/L for MMA). The optimum decision point for Hcy (i.e., 15 *μ*mol/L), however, falls well within the Hcy band (13.6-19.6 *μ*mol/L) [27].

Several studies have compared different markers for detecting B12 deficiency. These studies differed in the investigated cohorts and the reference used to define B12 deficiency. The majority of studies used one or two metabolic markers as the gold standard for the diagnosis of B12 deficiency, i.e., MMA or MMA with Hcy. This approach allows for comparisons between only the diagnostic characteristics of the direct markers HoloTC and B12. A comparison of the characteristics of the direct markers to those of metabolic markers is not possible in such a study setting. Further, the approach of using metabolic markers as a reference for the definition of B12 deficiency has been criticized due to their nonspecificity [11, 13].  Table 6 summarizes the findings of several studies employing only functional markers [46–49] as well as other more complex markers for definition of B12 deficiency (i.e., red cell cobalamin [43], Fedosov's *ω* [25], the predecessor of 4cB12). Interestingly, in the studies employing more complex markers for definition of B12 deficiency, HoloTC was better than MMA (AUC 0.78 [43] and 0.916 [25]) and Hcy (AUC 0.79 [43] and 0.872 [25]) in predicting B12 deficiency. Of note, the AUC's found in our study are well comparable with those reported by Fedosov [25].

It remains unclear why HoloTC is superior to the other markers for recognizing B12 deficiency in women 50 years and older. It is possible that because the present study did not employ the folate correction for 4cB12 age- and gender-specific effects could have been introduced [27]. Of the investigated samples, 3,614 had a concomitant folate analysis. The subanalysis of these samples by ROC analysis yielded identical conclusions for the different markers with regard to the detection of 4cB12<−0.5 in the whole cohort and in the different subgroups, especially also in women 50 years and older (data not shown). Other factors that could explain the gender difference include hormonal changes after menopause and that a fraction of the women may have taken postmenopausal hormone replacement therapy (HRT). Since estrogens influence total B12 (but not HoloTC) levels via decreased haptocorrin expression (and thus total decreased B12 concentrations), menopause and HRT could introduce a bias that diminishes the diagnostic accuracies of total B12 in women by reducing the sensitivity for detection of vitamin B12 deficiency at a given cutoff [34, 36, 50–52]. The observation that the AUC of B12 (but not of HoloTC) in women 50 years and older is lower than the AUC of women younger than 50 years (Table 4) supports this hypothesis. A further potential bias could have been introduced by pregnancy that was not disclosed to the laboratory. When we restricted the analysis to women aged 45 to 49 (*n* = 604), an age range in which pregnancy is very rare, we observed practically identical AUCs to those for the subgroup of all women < 50 years (data not shown). This suggests that pregnancy is not a likely cause of the differences in the marker characteristics among the different age groups.

Our study has strengths and limitations. The main limitation is that the investigated samples were not clinically well described [11, 13, 53]. Data, such as the hemoglobin concentration, vitamin B6 concentration, renal function, neurological deficits, anemia, and cognition, as well as B12 intake, are lacking. This lack of data makes a detailed and conclusive analysis of the differences in the diagnostic accuracies impossible. However, 4cB12, as the reference index used for the diagnosis of B12 deficiency, requires only the direct and metabolic markers of B12 deficiency, age, and folate when diagnosing B12 deficiency. Aside from the limitation of the folate data, which was available in only a subfraction of the investigated samples, all required markers were available for the determination of 4cB12. We consider 4cB12 a better standard than the metabolic markers MMA and Hcy. However, a reversal of symptoms and a correction of markers as a result of B12 supplementation would be the ideal marker for the definitive confirmation of B12 deficiency [54]. Further, haptocorrin deficiency could have biased our B12 results. Since we did not measure haptocorrin, we cannot assess the impact of haptocorrin deficiency on our results [55]. Finally, it could be argued that an analysis should be done on the patients rather than on the samples. We chose our approach in order to have a more robust database, not at last because it reflects clinical routine better just than one time measurements on each patient. However, we repeated the analyses in a database only containing the first measurement of every patient and provide the results in supplementary Tables [Supplementary-material supplementary-material-1][Supplementary-material supplementary-material-1]. We could demonstrate that the baseline characteristics ([Supplementary-material supplementary-material-1]) as well as the cutoffs to detect low B12 status (Tables [Supplementary-material supplementary-material-1] and [Supplementary-material supplementary-material-1]) are comparable. Further, we could also confirm the finding that HoloTC has a higher diagnostic accuracy than B12 because of its superiority in women aged 50 years or more (Tables [Supplementary-material supplementary-material-1] and [Supplementary-material supplementary-material-1]). A strength of the current study is the large sample size. To our knowledge, this is the largest analysis of different markers for identifying a decreased 4cB12 status to date. In summary, we believe that these limitations do not invalidate our findings.

This retrospective analysis was performed with a large cohort of patients who were referred for the investigation of B12 deficiency. In using 4cB12 as a novel index marker of B12 status, we were able to confirm cutoffs used for diagnosis of subclinical and possible/potential B12 deficiency. HoloTC was the better of the two direct markers, and MMA was the better of the two metabolic markers for detecting B12 deficiency. HoloTC had the highest diagnostic accuracy among these 4 markers, which was driven by the superiority of HoloTC in women 50 years or older. In younger women and in men, MMA and B12 were equivalent to HoloTC for the detection of B12 deficiency. Considering the costs (MMA > HoloTC > Hcy > B12), a stratified, stepwise approach is recommended for investigations of B12 deficiency. The present study offers cutoffs for potential use in such an approach. For women younger than 50 years of age and for men, B12 seems to be the first-line marker of choice, whereas for women 50 years and older, HoloTC seems to be a preferable first-line marker for the identification of B12 deficiency.

## Figures and Tables

**Table 1:** Baseline characteristics of the 11,833 samples.

Parameter (units)	Median (IQR or %)
Patient age (years)	56 (41, 68)
Female gender (*n*)	58.8% (57.9, 59.7) (6961)
Holotranscobalamin (pmol/L)	64 (45, 93)
B12 (pmol/L)	292 (222, 393)
MMA (nmol/L)	175 (137, 234)
Hcy (*μ*mol/L)	12.1 (9.7, 15.3)
HoloTC/B12 % (%)	21.1% (15.7, 27.9)
Haptocorrin-bound B12 (pmol/L)	226 (164, 312)
4cB12	0.24 (-0.11, 0.58)
Elevated B12 (4cB12 > 1.5) (*n*)	0.9% (0.75, 1.1) (107)
B12 adequacy (4cB12: -0.5 to 1.5) (*n*)	90.4% (89.9, 91) (10701)
Low B12 (4cB12: -1.5 to -0.51) (*n*)	8.2% (7.7, 8.7) (967)
Possible B12 deficiency (4cB12: -1.51 to -2.5) (*n*)	0.43% (0.33,0.57) (51)
Probable B12 deficiency (4cB12<−2.5) (*n*)	0.06% (0.03, 0.12) (7)

IQR: interquartile range for continuous variables; CI: confidence interval for proportion. Both are given in brackets.

**Table 2:** Marker cutoffs for detecting subclinical B12 deficiency (4cB12≤ -0.5 and >-1.5) with a sensitivity or specificity of 99% at a given specificity and sensitivity, as well as the optimum decision point.

Marker	Cutoff 99% sensitivity	Corresponding specificity (%)	Cutoff 99% specificity	Corresponding sensitivity (%)	Optimum decision point	Corresponding sensitivity/specificity (%)
HoloTC (pmol/L)	<73	44.1	<25	27.5	<45	85.7/81.2
B12 (pmol/L)	<351	36.7	<142	28.2	<229	86.1/77.7
MMA (nmol/L)	>152	37.8	>480	29.3	>245	81.8/83.4
Hcy (*μ*mol/L)	>8	12.6	>29	11.5	>15	67.7/76.7

**Table 3:** Marker cutoffs for detecting possible or probable B12 deficiency (4cB12≤ -1.5) with a sensitivity or specificity of 99% at a given specificity and sensitivity, as well as the optimum decision point.

Marker	Cutoff 99% sensitivity	Corresponding specificity (%)	Cutoff 99% specificity	Corresponding sensitivity (%)	Optimum decision point	Corresponding sensitivity/specificity (%)
HoloTC (pmol/L)	<56.5	60	<19	77.6	<27	93.1/96
B12 (pmol/L)	<320	41.6	<115	56.9	<167	94.8/92.3
MMA (nmol/L)	>158	39.1	>723	72.4	>466	94.8/96.4
Hcy (*μ*mol/L)	>6.2	3	>34	32.8	>16,4	87.9/80.9

**Table 4:** Diagnostic accuracies of the different markers for detecting low B12 (4cB12< -0.5 and >-1.5) in men and women over or under 50 years.

Marker (*N* whole group) (*n* with low 4cB12)	Women (*N* = 6, 961) (*n* = 564)	Men (*N* = 4, 872) (*n* = 403)	<50 years (*N* = 4, 676) (*n* = 274)	≥50 years (*N* = 7, 157) (*n* = 693)	Women, <50 years (*N* = 3, 202) (*n* = 195)	Women, ≥50 years (*N* = 3, 759) (*n* = 369)	Men, <50 years (*N* = 1, 474) (*n* = 79)	Men, ≥50 years (*N* = 3, 398) (*n* = 324)
HoloTC	0.919^∗^	0.913	0.919	0.918^∗^	0.923	0.925 ^∗^	0.915	0.910
B12	0.898	0.91	0.923	0.893	0.918	0.887	0.936	0.899
MMA	0.913	0.902	0.921	0.899^†^	0.925	0.905^†^	0.911	0.895
Hcy	0.783^‡^	0.831^‡^	0.771^‡^	0.799^‡^	0.773^‡^	0.793^‡^	0.855^‡^	0.817^‡^

^∗^Significant difference between the AUC of B12 and HoloTC (*p* < 0.05).^†^Significant difference between HoloTC and MMA (*p* < 0.05). ^‡^Significant difference between Hcy and all other markers (*p* < 0.01). The difference in the AUC of B12 and MMA was not significant in either subgroup (*p* = 0.07 in women; *p* = 0.09 in women ≥50 years).

**Table 5:** Diagnostic accuracies of the different markers for detecting potential or probable B12 deficiency (4cB12< -1.5) in men and women over or under 50 years.

Marker (*N* whole group) (*n* with 4cB12< -1.5)	Women (*N* = 6,961) (*n* = 28)	Men (*N* = 4, 872) (*n* = 30)	<50 years (*N* = 4, 676) (*n* = 9)	≥50 years (*N* = 7, 157) (*n* = 49)	Women, <50 years (*N* = 3, 202) (*n* = 5)	Women, ≥50 years (*N* = 3, 759) (*n* = 23)	Men, <50 years (*N* = 1, 474) (*n* = 4)	Men, ≥50 years (*N* = 3, 398) (*n* = 26)
HoloTC	0.979	0.986	0.977	0.983	0.989	0.978	0.97	0.987
B12	0.954	0.982	0.931	0.974	0.878	0.971	0.996	0.977
MMA	0.972	0.989	0.997	0.973	1	0.957	0.995	0.987
Hcy	0.887^#^	0.912^‡^	0.756^#^	0.916^‡^	0.747	0.9°	0.746	0.936^‡^

^‡^Significant difference between Hcy and all other markers (*p* < 0.01). ^#^Significant difference between Hcy and HoloTC or MMA (*p* < 0.05). °Significant difference between Hcy and HoloTC or B12 (*p* < 0.05).

**Table 6:** Summary of studies comparing the diagnostic accuracy of HoloTC and B12 to diagnose B12 deficiency.

Author	Year	Number and condition of studied subjects	Definition of B12 deficiency	AUC HoloTC (95% CI)	AUC B12 (95% CI)
Hermann and Obeid [47]	2003	204 99 healthy subjects and 111 vegetarians	MMA > 270 nmol/L	0.88 (n.a.)	0.84 (n.a.)
Hvas and Nexo [48]	2005	806 clinical study participants with creatinine <120 *μ*mol/L	MMA > 750 nmol/L and Hcy > 15 *μ*mol/L	0.90 (0.83, 0.97)	0.85 (0.77, 0.94)
Schrempf et al. [49]	2011	851 neurological patients with normal creatinine (males ≤106 *μ*mol/L; females ≤80 *μ*mol/L)	MMA ≥ 470 nmol/L	0.72 (0.65, 0.78)	0.66 (0.51, 0.82)
Herrmann and Obeid [46]	2013	1034 laboratory patient referrals with creatinine <97.2 *μ*mol/L	MMA > 750 nmol/L	0.71 (n.a.)	0.63 (n.a.)
Fedosov [25]	2010	865 pooled participants of three different studies	*ω*≤−0.516	0.928 (n.a.)	0.881 (n.a.)
Valente et al. [43]	2011	700 elderly memory clinic patients	Red cell B12 < 33 pmol/L	0.90 (0.86, 0.93)	0.80 (0.75, 0.85)
Present study	2020	11,833 samples referred to laboratory	4cB12<−0.5	0.912 (0.91, 0.92)	0.899 (0.89, 0.91)

CI: confidence interval, n.a.: not available.

## Data Availability

The data used to support the findings of this study are available from the corresponding author upon request.

## Supplementary Materials

Supplementary Materials The 5 supplementary tables contain the following legends: Supplementary Table S1: baseline characteristics of the 9,464 patients. Supplementary Table S2: marker cutoffs for detecting subclinical B12 deficiency in 9,464 patients. Supplementary Table S3: marker cut-offs for detecting possible or probable B12 deficiency in 9,464 patients. Supplementary Table S4: diagnostic accuracies of the different markers for detecting low B12 (4cB12<−0.5 and >-1.5) in 9,464 patients. Supplementary Table S5: diagnostic accuracies of the different markers for detecting potential or probable B12 deficiency (4cB12<−1.5) in 9,464 patients.
