# Supplementary material for: Diagnostic Accuracy of Holotranscobalamin, Vitamin B12, Methylmalonic Acid, and Homocysteine in Detecting B12 Deficiency in a Large, Mixed Patient Population
Source: Dis Markers. 2020 Feb 7;2020:7468506. doi: 10.1155/2020/7468506 (PMC7017578; doi:10.1155/2020/7468506)
Supplement: Supplementary Materials — The 5 supplementary tables contain the following legends: Supplementary Table S1: baseline characteristics of the 9,464 patients. Supplementary Table S2: marker cutoffs for detecting subclinical B12 deficiency in 9,464 patients. Supplementary Table S3: marker cut-offs for detecting possible or probable B12 deficiency in 9,464 patients. Supplementary Table S4: diagnostic accuracies of the different markers for detecting low B12 (4cB12<−0.5 and >-1.5) in 9,464 patients. Supplementary Table S5: diagnostic accuracies of the different markers for detecting potential or probable B12 deficiency (4cB12<−1.5) in 9,464 patients. [file 7468506.f1.pdf]

Supplementary materials to:

Diagnostic accuracy of holotranscobalamin, vitamin B12, methylmalonic acid, and homocysteine in detecting B12 deficiency in a large, mixed patient population

Running title: Markers of B12 deficiency in a mixed cohort

Araceli Jarquin Campos [a,b], Lorenz Risch [b,c,d], Urs Nydegger [b], Jacobo Wiesner [a], Maclovia Vazquez Van Dyck [a], Harald Renz [e], Zeno Stanga [f,g], Martin Risch [b,h]

[a] Universidad Autonòma de Guadalajara, Faculdad de Medicina, Zapopan, Jalisco, Mexico

[b] Labormedizinisches zentrum Dr. Risch, Vaduz, Liechtenstein

[c] Center of Laboratory Medicine, University Institute of Clinical Chemistry, University of Bern, Bern, Switzerland

[d] Private University of the Principality of Liechtenstein, Triesen, Liechtenstein

[e] Institute of Laboratory Medicine, Philipps University Marburg, Marburg, Germany

[f] Department for Diabetes, Endocrinology, Nutritional Medicine and Metabolism, Bern University Hospital and University of Bern, Bern, Switzerland

[g] Swiss Academy of Military and Disaster Medicine, Ittigen, Switzerland

[h] Zentrallabor, Kantonsspital Graubünden, Chur, Switzerland

Supplementary tables: 5

Correspondence:
Martin Risch, MD
Kantonsspital Graubünden
Zentrallabor
Loësstrasse 170
7000 Chur
Tel. + 41 81 256 65 31
Fax + 41 81 256 65 28
Email martin.risch@ksgr.ch

In the following tables we report on the results on a per patient analysis of the dataset. Only the data obtained at the occasion from the first blood drawing were taken for this analysis, which included a total of 9,464 patients.

Table S1. Baseline characteristics of the 9,464 patients. IQR=interquartile range for continuous variables; CI= confidence interval for proportion. Both are given in [brackets].

**Parameter (units)**	**Median, IQR or 95%-CI**
Patient age (years)	54 [39,68]
Female gender (n)	59.3% [58.3,60.3] (5616)
Holotranscobalamin (pmol)	64 [45,93]
B12 (pmol/L)	286 [217,381]
MMA (nmol/L)	177 [137,237]
Hcy (μmol/L)	12.2 [9.7,15.3]
HoloTC/B12 (%)	21.6% [16,28.4]
Haptocorrin-bound B12 (pmol/L)	220 [160,300]
4cB12	0.23 [-0.12,0.58]
-Elevated B12 (4cB12>1.5) (n)	0.89% [0.72, 1.1] (84)
- B12 adequacy (4cB12: -0.5 to 1.5) (n)	89.7% [89,90.3] (8486)
- Low B12 (4cB12: -1.5 to -0.51) (n)	8.9% [8.3,9.5] (841)
- Possible B12 deficiency (4cB12: -1.51 to -2.5) (n)	0.5% [0.37,0.66] (47)
- Probable B12 deficiency (4cB12 < -2.5) (n)	0.06% [0.03;0.14] (6)

Table S2. Marker cut-offs for detecting subclinical B12 deficiency (4cB12 ≤-0.5 and >-1.5) with a sensitivity or specificity of 99% at a given specificity and sensitivity, as well as the optimum decision point in 9,464 patients.

**Marker**	**Cut-off 99% sensitivity**	**Corresponding specificity (%)**	**Cut-off 99% specificity**	**Corresponding sensitivity (%)**	**Optimum decision point**	**Corresponding Sensitivity/ Specificity (%)**
HoloTC (pmol/L)	<72.4	44.2	<24.8	25.9	<45	85.2 / 81
B12 (pmol/L)	<352	34.4	<141	27.6	<229	87 / 76.6
MMA (nmol/L)	>153	39.6	>485	27.4	>238	83.7 / 82
Hcy (μmol/L)	>8	12.6	>29.5	11.1	>15	67.9 / 77.3

Table S3. Marker cut-offs for detecting possible or probable B12 deficiency (4cB12≤-1.5) with a sensitivity or specificity of 99% at a given specificity and sensitivity, as well as the optimum decision point in 9,464 patients.

**Marker**	**Cut-off 99% sensitivity**	**Corresponding specificity (%)**	**Cut-off 99% specificity**	**Corresponding sensitivity (%)**	**Optimum decision point**	**Corresponding Sensitivity/ Specificity (%)**
HoloTC (pmol/L)	<38.5	83.3	<19.5	76	<27	94 / 95.8
B12 (pmol/L)	<197	82.8	<114	56	<167	96 / 91.7
MMA (nmol/L)	>157	38.1	>736	74	>532	96 / 97.5
Hcy (μmol/L)	>6.1	2.6	>34.2	28	>16,4	90 / 80.7

Table S4. Diagnostic accuracies of the different markers for detecting low B12 (4cB12 <-0.5 and >-1.5) in 9,464 men and women over or under 50 years. * Significant difference between the AUC of B12 and HoloTC (p<0.05);† significant difference between HoloTC and MMA (p<0.05); ‡ significant difference between Hcy and all other markers (p<0.01); °significant difference between MMA and B12 (p<0.05). The difference in the AUC of HoloTC and B12 (p=0.056 in women) and HoloTC and MMA (p=0.087 in men ≥50 years) was not significant in either subgroup.

**Marker**<br>(N whole group)<br>(n with low 4cB12)	**Women**<br>(N=5589)<br>(n=475)	**Men**<br>(N=3822)<br>(n=344)	**<50 years**<br>(N=3876)<br>(n=244)	**≥50 years**<br>(N=5537)<br>(n=675)	**Women, <50 years**<br>(N=2630)<br>(n=171)	**Women, ≥50 years**<br>(N=3959)<br>(n=304)	**Men, <50 years**<br>(N=1246)<br>(n=73)	**Men, ≥50 years**<br>(N=2576)<br>(n=271)
HoloTC	0.911	0.912	0.914	0.914 *	0.918	0.919 *	0.914	0.909
B12	0.896	0.906	0.924	0.887	0.921	0.882	0.931	0.894
MMA	0.915 °	0.9	0.924	0.898 †	0.927	0.909 °	0.916	0.889
Hcy	0.784 ‡	0.825 ‡	0.769 ‡	0.80 ‡	0.769 ‡	0.799 ‡	0.851 ‡	0.808 ‡

Table S5. Diagnostic accuracies of the different markers for detecting potential or probable B12 deficiency (4cB12 <-1.5) in men and women over or under 50 years. * Significant difference between the AUC of B12 and HoloTC (p<0.05); # significant difference between Hcy and B12 and HoloTC (p<0.05); ^ significant difference between Hcy and B12 and MMA (p<0.05) ; ‡ significant difference between Hcy and all other markers (p<0.05).

**Marker**<br>(N whole group)<br>(n with 4cB12<-1.5)	**Women**<br>(N=5616)<br>(n=26)	**Men**<br>(N=3848)<br>(n=24)	**<50 years**<br>(N=3884)<br>(n=5)	**≥50 years**<br>(N=5580)<br>(n=43)	**Women, <50 years**<br>(N=2634)<br>(n=4)	**Women, ≥50 years**<br>(N=2982)<br>(n=22)	**Men, <50 years**<br>(N=1250)<br>(n=3)	**Men, ≥50 years**<br>(N=2598)<br>(n=21)
HoloTC	0.993 *	0.98	0.971	0.99*	0.99	0.995 *	0.956	0.983
B12	0.975	0.978	0.988	0.972	0.988	0.972	0.994	0.971
MMA	0.973	0.989	0.998	0.972	1	0.96	0.997	0.985
Hcy	0.895 #	0.932 ‡	0.831	0.905 ‡	0.748	0.893 #	0.94 ^	0.924 ‡
